# Progression of oncolytic virus in liver cancer treatment

**DOI:** 10.3389/fonc.2024.1446085

**Published:** 2024-09-26

**Authors:** Xuesi Hua, Siyu Xuan, Yangyang Tang, Shilin You, Shang Zhao, Ye Qiu, Yinqing Li, Yongqing Li, Yanping Su, Peng Qu

**Affiliations:** ^1^ School of Dentistry, University of Michigan, Ann Arbor, MI, United States; ^2^ Department of Histology and Embryology, Shandong First Medical University & Shandong Academy of Medical Sciences, Jinan, Shandong, China; ^3^ Department of Pharmacy, Changchun University of Traditional Chinese Medicine Innovation Practice Center, Changchun, Jilin, China; ^4^ Institute of Animal Husbandry and Veterinary Medicine, Beijing Academy of Agricultural and Forestry Sciences, Beijing, China; ^5^ Department of Pharmacy, Zhejiang University of Technology Fuyang Yinhu Institute of Innovation and Entrepreneurship, Hangzhou, Zhejiang, China

**Keywords:** HCC, oncolytic virus, influenza virus, herpes simplex virus, adenovirus

## Abstract

The liver plays a crucrial role in detoxification, metabolism, and nutrient storage. Because liver cancer ranks among the top three leading causes of death globally, there is an urgent need for developing treatment strategies for liver cancer. Although traditional approaches such as radiation, chemotherapy, surgical removal, and transplantation are widely practiced, the number of patients with liver cancer continues to increase rapidly each year. Some novel therapeutics for liver cancer have been studied for many years. In the past decade, oncolytic therapy has emerged, in which viruses selectively infect and destroy cancer cells while sparing normal cells. However, oncolytic virotherapy for liver cancer remains relatively obscure due to the aggressive nature of the disease and the limited effectiveness of treatment. To keep pace with the latest developments in oncolytic tumor therapy for liver cancer, this review summarizes basic science studies and clinical trials conducted within 5 years, focusing on the efficacy and safety profiles of the five most commonly used oncolytic viruses: herpes simplex virus, adenovirus, influenza virus, vaccinia virus, and coxsackievirus.

## 1 Introduction

As of 2020, liver cancer stands as the third leading cause of mortality worldwide, claiming the lives of 830,200 individuals annually (1). The number of liver cancer diagnoses and deaths is projected to increase by 55% from 2020 to 2040 (2). In the United States alone, liver cancer incurs an annual cost of $454.9 million, averaging $32,907 per patient. This includes the cost of healthcare and loss of productivity due to liver disease. Hepatocellular carcinoma (HCC) is one of the most common types of liver cancer, accounting for 90% of primary liver cancers (3). Without adequate treatment, patients infected with viruses that cause hepatitis can progress into chronic liver disease, predisposing them to HCC. Other risk factors include alcohol abuse, obesity, fatty liver, and diabetes (4). Diagnosis of HCC follows the Barcelona Clinic Liver Center (BCLC) strategy, which guides treatment decisions at different disease stages (5). In the early stages (BCLC 0-A), treatment primarily includes surgical resection and ablation (6). For intermediate cases (BCLC B), conventional transarterial chemoembolization improved survival rate (7). As the disease progresses to advanced stages (BCLC C), patients manifest cancer-related symptoms, prompting the utilization of sorafenib, a tyrosine kinase inhibitor, approved by the Food and Drug Administration (8). However, in the terminal stage (BCLC D), therapeutic options become severely limited. Liver transplantation emerges as a potentially viable intervention, but its scientific efficacy remains unproven (9). Traditional liver cancer treatments, such as immunotherapies, and transarterial chemoembolization, have not shown great effectiveness due to the immune tolerance of the liver, and not all patients are eligible for these treatments (6). Thus, new therapeutic methods are urgently needed.

Cancer is the leading cause of death in every country in the world (10). Since 1921, when cancer cells first appeared, humans have sought treatments to improve survival rates. Since entering the 21st century, genetic engineering technology has made continuous progress and its application in medicine has been greatly developed, among which oncolytic virus therapy stands out among many cancer treatment methods (11). Oncolytic viruses (OVs) are genetically engineered viruses that specifically fight cancer cells. It can recognize and infect different cells in the tumor environment, replicate in tumor cells through different regulatory mechanisms, lyse tumor cells, and be released from tumor cells to further infect surrounding tumor cells; while in normal cells, oncolytic The virus is cleared by the body’s immune system without affecting its normal growth (12). Since the discovery of using OVs to treat cancer cells, preclinical studies and clinical trials have employed OVs in HCC and have demonstrated some progress (13).

The effectiveness of OVT against HCC can vary due to several factors, such as changes in receptor expression, host immune response, TME, and genetic alterations (14). Commonly used virus vectors for HCC OVT include HSV, ADV type 5, IV, oncolytic VV, and COX-A, etc. This review summarizes preclinical studies from 2022 to 2024 and clinical trials from 2015 to 2024 to investigate the OVs in HCC treatment. Common administration routes include intravenous, intrasplenic, intratumoral, intraarterial, intrabiliary, etc (15).

## 2 Oncolytic viruses

### 2.1 Mechanisms for genetically engineered oncolytic viruses

Oncolytic viruses (OVs) have emerged as a promising approach in cancer therapy, leveraging the natural ability of viruses to selectively target and destroy tumor cells while leaving healthy ones unaffected. There are three primary mechanisms to genetically engineer OVs:

#### 2.1.1 Type I interferon signaling pathway regulation

To achieve antitumor activity, one of the most common ways that OVs use is to downregulate the IFN signaling pathway, making tumor cells more susceptible to the OVs that will then replicate and kill the tumor cell through direct lysis (16, 17). This process is primarily driven by the susceptibility of oncolytic viruses (OVs) to interferon (IFN) and the decreased responsiveness of tumor cells to IFN. Preclinical studies using vesicular stomatitis virus for HCC showed that by IFN signal acts like a cytokine to direct the priming of virus and tumor-reactive T cells, which induces oncolysis and host immune response (18).

#### 2.1.2 Tumor-specific promoters

Tumor or tissue-specific gene promoters are engineered into the OVs to selectively transcribe targeted gene sequences. This allows for rapid replication within tumor cells while limiting replication in normal cells (19). Conventionally, homologous recombination technique has been used. However, this method has been limited by its low efficacy and the complication of multiple steps involved (20). To solve this problem, several approaches have been used to insert tumor-specific promoters to OVs. The CRISPR-Cas9 system was introduced. By using a guide RNA to direct the Cas9 enzyme to a specific DNA site, it allows a donor DNA template containing the new promoter to be integrated via homology-directed repair. Yuan et al. showed that CRISPER-Cas9 system induces higher efficiency of homologous recombination by 3% when introducing DsRed into oADV (21). Additionally, Terada et al. used a bacterial artificial chromosome (BAC) -based model, in which the backbone of BAC can effectively exchange with the promoter of interest through sequential, site-specific recombination, to express luciferase protein by inserting various viral promoters on oHSV (22). Gateway recombination cloning was effectively used insert-expression vector, M134, GOI, and M136 with eGFP as fluorescent marker, into myxoma oncolytic virus (23). Moreover, to identify the site of insertion, transposon insertion strategy has been largely used to scan the genome nonprejuidicely. Kretschemer et al. used Tn7 transposon to find several sites for promoter-based expression insertions in the oADV genome, and those approaches have been proved to perform easily and effectively (24).

#### 2.1.3 Gene silencing

Certain viral genes necessary for replication in normal cells, but not required by tumor cells, are deleted. This allows viruses to replicate rapidly within tumor cells with attenuated replicability in normal cells (25). Double-stranded interfering RNAs (RNAi) can guide Argonaute proteins to target tumor cell RNAs via Watson-Crick base-pairing to achieve gene silencing within the tumor.

Importantly, OVs contain genetic sequences not only for mediating replication but also for modifying the tumor immune microenvironment (TME) (16). Alterations in the TME can provoke innate and adaptive immune responses and inhibit tumor angiogenesis, leading to tumor death (26). Although this may initially limit the spread of OVs in tumor cells, the cell lysis induced by viruses and the danger-associated molecular patterns triggered by OVs can overcome immunosuppression and promote antitumor immunity (27). To prevent the spread of OVs into healthy cells, neutralizing antibodies and cytokines produced in response to viruses initiate immune reactions. However, the clinical application of OVs in cancer therapy is challenging, particularly regarding their toxicity and pathogenicity to humans. Addressing these challenges is crucial for the broader adoption and effectiveness of OV-based cancer therapies (19).

### 2.2 Antitumor mechanisms of OVs

**Figure 1:**
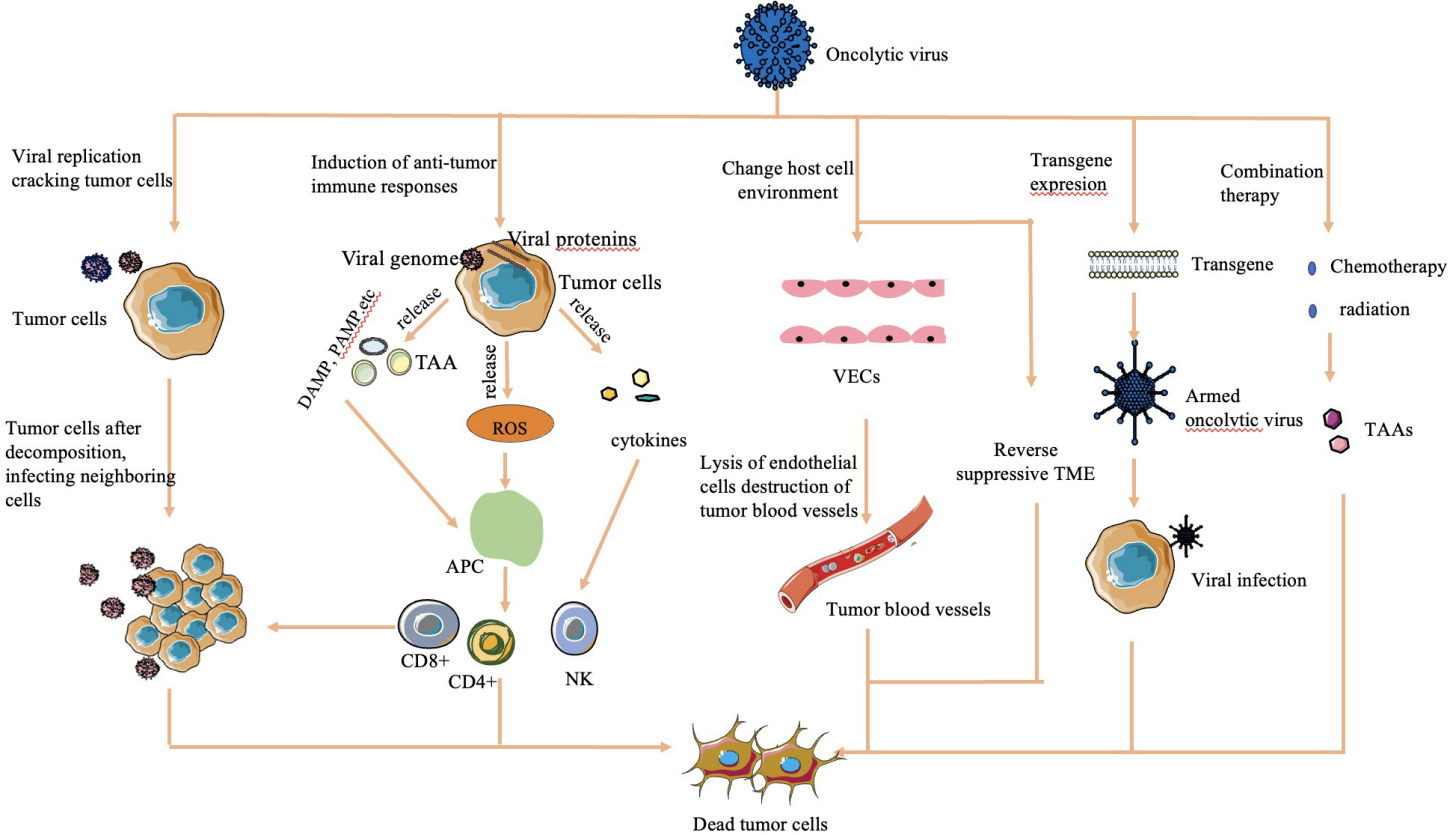
Mechanisms of cell lysis used by oncolytic viruses (16, 30, 32). The mechanism of OV cell lysis can be categorized into major categories. 1) Direct lysis due to a large volume of virus by replication. 2) Cytotoxicity by proteins encoded by the virus, which leads to tumor cell apoptosis and autophagy death. 3) Anti-tumoral immunity that leads to induction of host immune response, escape of the virus from the host response, and release of TAAs to act on adjacent sites. 4) Sensitization of chemotherapy and radiation. 5) Transgene expression through genetic engineering. 6) Change in host cell environment, including reversal of host TME and destruction of tumor blood vessels.

The mechanisms by which OVs effectively kill tumor cells are diverse and multifaceted (**Figure 1**):

#### 2.2.1 Direct lysis

OVs overwhelm tumor cells with the production of viruses, causing direct lysis when the viral load exceeds the capacity of tumor cells to contain them (28).

#### 2.2.2 Transgene expression

Genetically engineered OVs can express transgenes that induce cytotoxic effects, leading to tumor cell apoptosis and autophagy (29).

#### 2.2.3 Sensitization to chemotherapy and radiation therapy

OVs sensitize tumor cells to chemotherapy and radiation therapy, enhancing their effectiveness in killing tumor cells (30).

#### 2.2.4 Antitumoral activity

OVs stimulate an antitumoral immune response by triggering cytokine release upon detection by the host immune system. This immune response targets virus-infected tumor cells through the innate pathway, causing release of tumor-associated antigens, further enhancing immune recognition and tumor cell death (16).

#### 2.2.5 Vasculature targeting

Some OVs are engineered to target the vasculature of tumor cells, reducing their blood supply and causing tumor regression (31).

#### 2.2.6 Alteration of TME

OVs can modify the immunosuppressive TME created by tumor cells, increasing the infiltration of antigen-presenting cells and immune cells into the tumor. This alteration helps restore the immune balance and enhances the immune response against the tumor (32).

These mechanisms collectively contribute to the potent antitumor effects of OVs.

Furthermore, the adaptability of OVs enables their potential incorporation into multimodal approaches to cancer treatment, presenting promising opportunities for enhancing patient outcomes.

Both clinical trials and preclinical studies have demonstrated the relative safety of oncolytic virotherapy (OVT), with minimal reported adverse effects. This safety profile underscores the potential of OVT as a groundbreaking treatment for cancer. Continued research and development in this field hold promise for further enhancing the efficacy and safety of OVs as a therapeutic approach against cancer (33).

### 2.3 Categories of OVs

The diversity of viruses being explored for OVT highlights the breadth of research in this field. Both natural and engineered viruses show promise as potential candidates for cancer treatment. Some viruses that are used for OVT include herpes simplex virus (HSV), adenovirus (ADV) type 5, influenza virus (IV), oncolytic vaccinia virus (VV), and coxsackievirus A (COX-A), measles virus, poliovirus, retrovirus, reovirus, parvovirus H1, vesicular stomatitis virus, Newcastle Disease virus, etc. (27). In recent years, nearly all of these viruses have been investigated in both preclinical basic science studies (**Table 1**) and clinical trials (**Table 2**) for liver tumor OVT. This underscores the extensive research being conducted to evaluate the efficacy and safety of viruses in targeting and destroying tumor cells, particularly in the context of liver cancer.

**Table 1:** Viruses used for liver tumor or liver metastasis treatment in basic science studies.

Virus Types	Product Names	Year	Modification	Model	Ref.
HSV	humanized scFv against human PD-1 (hPD-1scFv)	2022	Insertion of humanized hPD-1 blocker gene	Mouse	(42)
Morreton Virus	MORV, University of Texas	2023	Unmodified wildtype	Mouse	(108)
ADV	Ad-GD55–α-Tim-3	2023	Inhibition of T-cell TIM-3)	Mouse, *in vitro*	(54)
Newcastle Disease Virus (NDV)	Oncolytic virus M1	2022	Unmodified wildtype	Mouse, *in vitro*	(109)
Poxvirus	CF33	2023	Deletion of *J2R* (TK) gene and addition of human sodium iodide symporter (hNIS)	Mouse, *in vitro*	(110)
VV	oncoVV-AVL	2022	Expression of gene encoding *Aphrocallistes vastus* lectin	Mouse, *in vitro*	(111)
	VvDD-IL15Rα	2022	Expression of superagonist IL-15 and erastin plus the deletion of 2 viral genes that encode thymidine kinase and vaccinia growth factor	Mouse, *in vitro*	(71)
	OncoVV-AVL	2024	Expression of gene encoding *Aphrocallistes vastus* lectin	*in vitro* Mouse	(70)
IV	rFlu-huPD1	2022	PB1 fragment encodes the heavy chain of PD-1 antibody and polymerase acid protein fragment encodes PD-1 antibody light chain.	Mouse, *in vitro*	(112)
Measles Virus	MV	2023	Unmodified wildtype	*in vitro*	(113)
Reovirus	Reo	2018	Unmodified wildtype clinical grade oncolytic orthoreovirus	Mouse, *in vitro*	(36)
Alphavirus	M1-VCPI	2017	Expression of valosin-containing protein inhibitors (VCPIs)	Mouse, *in vitro*	(114)
	SINV-GM-CSF	2024	GM-CSF carrying Sindbis virus. Mutation (G to S) at amino acid 285 in the nsp1 protein	*in vitro* Mouse	(115)
COX-A21	V937	2024	Genetically unmodified Kuykendall strain of COX-A21	Mouse, *in vitro*	(87)

**Table 2:** Viruses used for liver tumor or liver metastasis treatment in clinical trials.

Virus	OVT Product	Year	Modification of Virus	Phase	Path	Ref.
HSV	NV1020, BioReliance	2010	Deletion of UL56 internal repeat gene and UL24 gene expression.	I/II	Herpetic artery infusion	(45)
ADV	OBP-301, Oncolys Biopharma Inc.	2023	Attenuated type 5 ADV with an *hTERT* promoter	I	Intertumoral Injection (IT)	(56)
VV	Pexa-Vec, JX-594, Biotherapeutics Inc. and Transgene S.A.	2019	Inactivated thymidine kinase to express human granulocyte-macrophage colony-stimulating factor (GM-CSF) and β-galactosidase	IIb	Intravenous (IV) infusion followed by IT	(73)
	VvDD (JX-929), Jennerex Biotherapeutics	2016	Deletion of vaccinia growth factor and TK	I	IV, IT	(116)
Vesicular Stomatitis Virus	VSV-IFNβ -TYRP1, Mayo Clinic	2023	Express IFN-β and Tyrosine Related Protein 1 (TYRP1)	I	IV, IT	(117)
COX-A21	V937, Viralytics	2023	Unmodified bioselected strain of CVA21	Ib	IV	(86)
Protoparvovirus H-1	ParvOryx	2021	Unmodified	II	IV, IT	(118)

Exploring the potential of multiple viruses allows researchers to identify the most effective candidates for OVT while considering safety, delivery methods, immune responses, etc. This comprehensive approach enhances our understanding of the diverse mechanisms that viruses use to exert oncolytic effects and paves the way for the development of novel and improved therapies for liver cancer and other malignancies.

In 2015, Talimogene laherparepvec (T-VEC), an HSV-1 derived JS1 OV strain, became the first and only OVT approved for clinical use by the Food and Drug Administration (34). T-VEC has been a significant development in OVT. Its approval marked a milestone in cancer treatment, particularly for melanomas. By leveraging the natural ability of HSV-1 to infect and kill cancer cells, T-VEC demonstrated promising efficacy in shrinking tumors and prompting immune responses against cancer cells.

Pexastimogene devacirepvec (Pexa-Vec), a VV with deletion of thymidine kinase, an enzyme in the DNA precursor pathway, was designed to restrict viruses to only attack tumor cells, particularly HCC cells (35, 36). Pexa-Vec expresses granulocyte-macrophage colony-stimulating factor to recruit dendritic cells through interferon cytokine expression, enhancing tumor infiltration (37). Although this phase III trial requires further optimization in HCC treatment, evidence from studies and clinical trials supports both the safety and efficacy of Pexa-Vec (36).

## 3 Novel finding in OVT against liver cancer

### 3.1 HSV

HSV is a double-stranded DNA virus (38). Its virion has four components: a DNA core, an icosapentahedral capsid, an amorphous protein coat tegument crucial for HSV infection, and a glycoprotein-bearing lipid bilayer envelope, from the inner to the outer surface (39, 40). It exists as HSV-1 and HSV-2, with HSV-2 commonly associated with sexually transmitted diseases and HSV-1 linked to infections of the oral cavity and skin. HSV-1 has been extensively used in OVT for HCC because it exhibits rapid host cell entry, efficient replication, binding to receptors broadly expressed in different types of human cells and tissues, and ability to stimulate a strong cellular and humoral immune response (41).

However, despite its potential, challenges must be addressed before HSV is widely used in clinical settings for OVT, including complexity of vector engineering, short-term stability issues, and risk of affecting normal tissue (41). Although a considerable number of preclinical studies have used HSV as the predominant OV in liver tumor treatment, in recent years, no clinical trial was successfully completed for HSV OVT targeting liver tumors. Clinical trials utilizing HSV for treating other cancers, including melanoma, lung cancer, solid tumors, breast cancers, and glioma, have been extensively investigated and have shown promising outcomes (**Table 3**). This indicates the potential for HSV in cancer therapy, but further research and development are needed to overcome the current challenges associated with its use in treating liver cancer.

**Table 3:** Clinical trials using HSV oncolytic therapy for all cancer types.

Cancer Type	Virus Product	Modifications	Year	Phase	Pathway of Delivery	Ref.
Non-Small Cell Lung Cancer	ADV/HSV-tk, Merck	Adenovirus-mediated expression of HSV thymidine kinase	2024	II	IT	(119)
Solid Tumors	HSV1716 (Seprehvir), Nationwide Children’s hospital	Deletion of ICP34.5 gene and maintenance of TK expression	2019	I	IV	(120)
Primary Central Nervous System Tumors	HSV G207, Aettis, Inc., University of Alabama	Deletion of γ134.5 gene and disability of *lacZ* insertion in U_L_39	2017	I	IT	(121)
Malignant Glioma	M032, Aettis, Inc., University of Alabama	Expression of IL-12	2016	I/II	IT	(122)
Recurrent Glioblastoma	CAN-3110 (rQNestin34.5v.2)	Expression of *ICP34.5* by nestin promoter	2023	I	IT	(123)
Melanoma	OrienX010	Expression of GM-CSF, deletion of ICP34.5 and ICP47, and inactivation of ICP6.	2022	Ib	IT	(124)
Soft Tissue Sarcoma of Trunk and Extremities	Talimogene laherparepvec (T-VEC)	Expression of GM-CSF and deletion of *ICP47* and *ICP 34.5* gene.	2021	Ib/II	IT	(125)
Breast Cancer	Talimogene laherparepvec (T-VEC), Amgen	Expression of GM-CSF and deletion of *ICP47* and *ICP 34.5* gene.	2021	I	IT	(126)
Malignant Pleural Mesothelioma	HSV1716, Virttu Biologics Limited	Deletion of *ICP 34.5* using strain 17+	2020	I/IIa	Intrapleural Injection (IP)	(127)
Pancreatic Cancer	HF10	Deletion of *UL43, UL49.5, UL55, UL56*, and latency-associated transcripts, and overexpression of *UL53* and *UL54*.	2018	I	IT	(128)

Recent research has focused on genetically engineering a tumor-selective oncolytic HSV (oHSV) to express a human single-chain variable fragment targeting human programmed cell death 1 (PD-1) in mouse and nonhuman primate models with human liver cells implanted subcutaneously (42). PD-1, an inhibitory receptor on lymphocytes, impedes T-cell recognition and attacks upon binding to programmed death ligand 1 (PD-L1) (43). By designing a single-chain variable fragment against humanized PD-1, researchers assessed the antitumor efficacy of oHSV. The ideal PD-1 blockade candidate was selected and verified in mouse and nonhuman primate models. Results showed that mice treated with anti-PD-1-modified OV developed long-term memory of T-cell responses and reduced immunotherapy resistance.

In nonhuman primates, a humanized antibody against PD-1, called hu17D5, was constructed after library screening. hu17D5 is a single-chain antibody with better affinity to PD-1. After administering hu17D5 to nonhuman primates, a significant T-cell immune response was observed (*p* < 0.01) (42). Subsequently, an OV was engineered to express the hu17D5 gene, naming it YST-oHSV. The results demonstrated that 72 h after YST-oHSV injection, the viability of HCC cells decreased by 90%, whereas normal cells remained unaffected (42). Additionally, the antitumor activity increased after YST-oHSV injection. When YST-oHSV was injected into mice with HCC, tumor growth was significantly inhibited, leading to increased survival rates and tumor regression. YST-oHSV treatment increased CD8+ cytotoxic T-cell rejuvenation and the number of CD8+ memory T cells. YST-oHSV demonstrated great safety in nonhuman primate models, with no serious adverse effects (AEs).

Inappropriate delivery routes often limit the efficacy of oHSV in OVT. To solve this problem, surface-engineering-technique-masked oHSV with a galactose-polyethylene-glycol (PEG) polymer chain (glycosylated-PEG-oHSV) was generated to direct viruses to tumor sites and limit off-target effects, especially to the brain (44). Although glycosylated-PEG-oHSV did not affect oHSV replication, it exhibited increased specificity to the asialoglycoprotein receptor, which is selectively expressed on the surface of HCC cells, in a mouse model. This leads to enhanced tumor penetration into the center of HCC cells and reduced accumulation in non-liver organs, such as the brain and lung. Additionally, glycosylated-PEG-oHSV decreased the level of HSV-neutralizing antibodies and T cells after infection. Furthermore, it increased the release of antitumor cytokines, leading to significant infiltration into the tumor, and thereby, limiting tumor growth (44). Propidium iodide staining validated the cytotoxic effect of oHSV to induce HCC apoptosis and necrosis. The efficacy of glycosylated-PEG-oHSV is dose-dependent, with optimal efficiency at 0.2 μM.

There has been a lack of HSV OVT clinical trials in the past years; however, a phase I/II study in 2010 published in *Hum Gene Ther* showed the antimetastasis ability of HSV in liver metastasis from colorectal cancer (45). In the study, scientists engineered NV1020, wild-type HSV-1 modified with the deletion of UL24 and internal repeat UL56 genes, which confers the ability to replicate in a less harmful manner. Then, the thymidine kinase gene was introduced to allow controlled infection. NV1020 was administered to 13 patients in phase I and 19 patients in phase II via hepatic artery injection weekly over four weeks. The results showed promising outcomes, with 50% exhibiting stable disease and one patient showing partial response following chemotherapy. Median time to progression was 6.4 months, and median overall survival (OS) was 11.8 months, with a 12-month survival rate of 47.2% (45). These findings underscored the efficacy of NV1020 in stabilizing liver metastasis. Regarding safety, AEs were primarily observed within 24 h post-infusion, with no grade 3 reactions reported. Most AEs were grade 1 and 2 reactions, such as nausea and myalgia, and were effectively managed with analgesics and other supportive measures. No virus was detected in serum or saliva samples, but HSV-1 was detected on the skin of two patients during monitoring. Despite these promising results, no subsequent publications regarding further phase II/III trials have emerged. This suggests that although the initial findings were encouraging, further research may be necessary to progress to later-stage clinical trials, and ultimately, determine the broader efficacy and safety profile of NV1020 in treating liver metastases or HCC.

### 3.2 ADV

ADV is a nonenveloped double-stranded DNA virus characterized by an icosahedral nucleocapsid. It belongs to the Adenoviridae family and is typically isolated from human adenoids (46). ADV primarily affects children because they have lower humoral immunity than adults (47). Based on its genome structure, ADV is categorized into 52 serotypes and 7 species (A–G) (48). ADV infection manifests in various forms, such as respiratory tract infection, keratoconjunctivitis, gastrointestinal manifestations, and urinary tract infection (47). Human ADV species C type 5 has been extensively studied in OVT because it evades pre-existing immunity (49). ADV demonstrates a remarkable capacity to target tumor cells through various receptors, such as Coxsackie and ADV receptor, integrins, CD46, desmoglein-2, and sialic acid (50). Besides its high safety profile, tumor selectivity, and immunogenicity, ADV stands out as an OVT candidate for its efficient gene delivery and transient expression (51). Specifically, ADV can infect both dividing and non-dividing cells, expanding its applicability to different tumor types, including HCC (52). ADV does not integrate its DNA into the host genome during replication, reducing the risk of insertional mutagenesis, which is a common concern with many other viruses (53).

Recent preclinical research demonstrated the modification of ADV as Ad-GD55–α-Tim-3. This engineered ADV expresses E1A, a protein for viral replication controlled by the GP73 promoter, and encodes an antibody gene of immunosuppressive T-cell immunoglobulin domain and mucin-domain molecule-3 (TIM-3) (54). TIM-3, an immune checkpoint expressed on the surface of Th1 cells to regulate macrophage activation, exhibited higher expression in HCC cells than in healthy cells. This was confirmed through immunohistochemistry and western blot analysis with a significance level of *p* < 0.05 (55). Ad-GD55–α-Tim-3 infection in HCC cells led to a decrease in pro-inflammatory cytokines, such as IL-1β and IL-6, and an increase in anti-inflammatory cytokines, such as IL-10, which fosters a less inflamed environment for viral replication. HCC cells with Ad-GD55–α-Tim-3 also showed less immunosuppressive factors, such as TGF-β and IDO, which increased the immune response of the host to target HCC cells (54). Although Ad-GD55–α-Tim-3 inhibited HCC cell growth, it did not significantly induce apoptosis compared with wild-type ADV. In a tumor xenograft HCC mouse model, treatment with Ad-GD55–α-Tim-3 resulted in higher Ki-67 antigen expression and increased CD4/CD8 cell number. Thus, Ad-GD55–α-Tim-3 inhibits tumor growth with no observed cytopathic changes in mouse organs.

In 2023, a phase I clinical trial tested an attenuated Ad5 with a human telomerase reverse transcriptase (*hTERT*) promoter. This virus, named OBP-31, maintains telomere length with expression occurring exclusively in liver cancer cells but not in healthy or differentiated cells, thereby increasing its tumor selectivity (56). This is achieved when the *hTERT* promoter interacts with an internal ribosome entry site, enhancing the replicability of OBP-301, specifically in cancer cells. OBP-301 then causes tumor cell destruction through direct lysis via viral replication and induces immune responses facilitated by the cytokines, tumor necrosis factor and IL-1 (57).

Eighteen patients with HCC were recruited, with a median time since HCC diagnosis of 3.24 years. Thirteen patients had stage C cancer according to the BCLC system, and all patients were classified as Child-Pugh class A. qPCR analysis revealed no detectable OBP-301 DNA in most patients after 24 h, and none showed positive OBP-301 DNA in blood or urine tests 14 d post administration, indicating the safety of OBP-301 in patients with HCC (56). However, no patient achieved a complete response or partial response. Fourteen patients were in the stable disease stage, whereas four were in the progressive disease stage. The mean duration of stable disease to disease control was 5.55 weeks, with a median time to progression of 8.10 weeks. The median OS was 26.00 weeks, and the average time for disease control was 4.21 weeks. CD8+ cell number increased by an average of 56.3% 4 weeks after OBP-301 injection. Overall, whereas OBP-301 demonstrated safety and elicited an immune response in patients with HCC, its efficacy in terms of disease control and survival outcomes was modest at best, suggesting the need for further investigation or combination therapies to enhance its therapeutic potential in patients with HCC.

In a recent phase I trial, ADV-5 was combined with hTERTRibozyme-expressing HSV thymidine kinase to target liver metastasis in patients with GI cancer (58). hTERTRibozyme specifically targeted hTERT, which is prominently expressed in HCC cells (59). Coupled with HSV thymidine kinase, hTERTRibozyme enhanced its cytotoxicity to HCC cells. The clinical trial involved 18 patients, with only 2 patients exhibiting stable disease after an 8-week regimen. Median progression-free survival (PFS) was 1.1 months, indicating limited clinical efficacy (58). Median OS was 6.2 months, and the maximum tolerated dose was 2 × 10^12^ viral proteins with higher doses failing to yield better clinical results, and no pharmacodynamic assessment was conducted. Virus DNA remained undetectable at significant levels after 72 h, with a median circulating virus half-life of 10.1 min. Due to the lack of efficacy, Ad5CRT is not ready to proceed to the next stage of clinical trials.

In addition to liver tumors, ADV has been widely used in OVT clinical trials for various other types of cancer. For instance, Ad5-yCD/mutTKSR39rep-hIL12 was used for prostate tumors (60), LOAd703 was used for pancreatic cancers (61), and Cretostimogene received the fast track and breakthrough designations for bladder cancers (62).

### 3.3 Oncolytic VV

VV, also known as smallpox, is a poxvirus characterized by a brick-shaped envelope and a 200-kb double-stranded DNA genome (63). Unlike many other viruses, VV does not require specific receptors for cell entry. Instead, it utilizes a protein-based entry-fusion complex or cooperates with endosomes for membrane fusion (64). Because VV does not enter the nucleus, it is easier to control its replication. VV replicates entirely within the cytoplasm of infected cells using its own DNA-encoded enzymes, avoiding competition with host cell DNA and circumventing the endomembrane system (65). Many antiviral agents can limit VV spread, including ST-246 and cidofovir (63). Cell lysis usually occurs <24 h after infection (66). VV elicits a robust T cell and antibody immune response and demonstrates a broad host cell tropism, making it a promising candidate for OVT (67). Other advantages of using VV in OVT include an efficient delivery system, stability upon intravenous administration or storage in powder or solution, and ability to encode transgenes (68).

The potent cytotoxic effect of VV was found to trigger the host immune response during HCC treatment. *Aphrocallistes vastus* lectin (AVL), a marine lectin commonly found in sponges and algae, was combined with VV to improve the cytotoxicity of VV in HCC cells through PI3K/Akt and MAPK/ERK pathways (69). Cells infected with siVV-AVL had significantly reduced viability compared with those infected with VV alone, and its antiproliferative efficacy increased progressively. VV-AVL-infected cells had 30-fold higher apoptosis than wild-type PBS control cells. Measurement of virus concentration in HCC cells demonstrated that VV-AVL upregulated 2′-5′-oligoadenylate synthetase-like protein, thereby enhancing VV DNA replication and resulting in significantly higher virus titers. VV-AVL infection significantly increased the expression of type I interferon, notably IFN-α and IFN-β, particularly 36- and 48-h post-infection (69). This increase was mediated through phosphorylated IFN regulatory factor 3 (IRF3). VV-AVL also suppressed antiviral factors, including 2′-5′-oligoadenylate synthetase, IL enhancer binding factor 3, and phospholipid scramblase 1. Consequently, VV-AVL could replicate within the host cell by activating mammalian sterile 20-like kinase without encountering host defenses. In a mouse model, 30-d postinjection, VV-AVL significantly inhibited tumor growth. Consistently, histological examination revealed a notable presence of broken nuclei in VV-AVL-infected cells. Additionally, Zhang et al. in 2024 confirmed the effectiveness of VV-AVL in liver tumor treatment (70). They further discovered the mechanism of oncoVV-AVL, which involves reprogramming hepatocellular carcinoma (HCC) metabolism to promote reactive oxygen species (ROS). ROS, in turn, enhance the replication of oncoVV-AVL and induce tumor cell apoptosis.

In 2022, Liu et al. aimed to combine the vaccinia virus (VV) with erastin to improve its oncolytic effectiveness in liver tumor treatment (71). Erastin is a ferroptosis activator that can induce cell death in liver, colon, and ovarian cancer cells. Since both VV and erastin have been proven to inhibit tumor growth, this study investigated whether combining vvDD (VV with the deletion of thymidine kinase and vaccine growth factor) and erastin could lead to superior antitumoral activity. The results showed that although 80% of the mice exhibited inhibition of tumor growth with erastin treatment alone, the combination of erastin and vvDD (vvDD-IL15-Rα) led to a 100% reduction in tumor volume and 60% tumor cell regression (71). None of the five mice treated with the combination developed new tumors 12 days after treatment, whereas the untreated mice showed 83% new tumor growth. This indicates the immune memory provided by vvDD-IL15-Rα. Immune markers, IFN-γ and TNF-α, and immune cells, CD86^+^CD11c^+^ and dendritic cells, were also higher in the vvDD-IL15-Rα group than vvDD or erastin alone group.

A randomized phase II clinical trial conducted in 2013 investigated the oncolytic effectiveness of JX-594 (Pexa-Vec) in liver cancer treatment (72). Pexa-Vec, a vaccinia virus with inactive thymidine kinase, expresses human granulocyte-macrophage colony-stimulating factor and β-galactosidase. Low or high doses of Pexa-Vec were injected into the liver tumor on days 1, 15, and 29. The Choi response rate and intrahepatic disease control rate showed no significant differences between the injected and non-injected liver tumors at either dose. However, the survival rate was significantly higher in the injected group (14.1 months) compared to the non-injected group (6.7 months) at either dose.

In 2019, a randomized multicenter phase IIB clinical trial evaluated the effectiveness of Pexa-Vec combined with Best Supportive Care (BSC) versus BSC treatment alone in patients with HCC who had failed sorafenib therapy (73). This study highlighted the efficacy and safety of Pexa-Vec in patients with HCC. The survival rate of patients treated with Pexa-Vec + BSC (median OS: 4.2 months) did not significantly differ from those receiving BSC alone (median OS: 4.4 months). Both Pexa-Vec + BSC and BSC alone had a high likelihood of inducing AEs. Anti-β-galactosidase antibodies were detected in 56% of the patients receiving Pexa-Vec + BSC, indicating significant viral replication in HCC cells (73). Virus detection from urine or throat swabs ceased after day 8, whereas 21% of the patients had virus in rectal swab samples. ELISPOT analysis demonstrated a significant increase in T cells after Pexa-Vec injection, particularly evident after 6 weeks. The most expressed tumor antigens were MAGE-A1 and MAGE-A3, suggesting that Pexa-Vec can induce a tumor-specific T-cell immune response. Overall, whereas Pexa-Vec showed promise in inducing a tumor-specific immune response and good safety profile, it did not translate into a significant improvement in overall survival or disease control rate in this study population.

However, when comparing the effectiveness of Pexa-Vec with Sorafenib, the most commonly prescribed medication for HCC treatment, versus Pexa-Vec alone, a phase II trial showed a 62% disease control rate with Pexa-Vec alone and 59% Pexa-Vec with sorafenib (74). The Pexa-Vec was well-tolerated. The high dose of Pexa-Vec showed greater OS (14.1 months) vs the lower dose (6.7 months). Due to the higher effectiveness and safety profile of Pexa-Vec, the next stage clinical trial is warranted.

Later, a phase III clinical trial from 2015 to 2019, conducted at 142 sites in 16 countries with 459 patients, evaluated the efficacy of Pexa-Vec plus sorafenib versus sorafenib alone in HCC patients (75). The median OS was 12.7 months in Pexa-Vec plus sorafenib compared to 14.0 months in the control group. Median TTP was 2.0 months versus 4.2 months; objective response rate was 19.2% versus 20.9%; and disease control rate was 50% vs 57.3%, respectively (75). As a result, the addition of Pexa-Vec to the traditional sorafenib approach failed to demonstrate clinical benefits in treating HCC, leading to the early termination of the trial. Moreover, the safety profile was less optimal in the Pexa-Vec plus sorafenib patients, with 53.7% reporting serious AEs compared to only 35.5% in the sorafenib-only group (75).

Several factors have been proposed to explain the failure (75). First, TK1 gene was inactivated during the construction of Pexa-Vec, preventing the synthesis of thymine nucleotides essential for the replication of the OV. Additionally, sorafenib was not administered until the complication of the entire Pexa-Vec therapy, and its immunosuppressive effect, combined with the delay, allowed time for tumor growth, leading to a shorter TTP. For future trials, the time to combine Pexa-Vec with sorafenib and the dose of the virus will be needed to optimize its therapeutic potential in HCC treatment.

The potential effectiveness of VV in OVT clinical trials was also evident in solid tumors (76), colorectal carcinoma (77), head and neck cancers (78), etc.

### 3.4 COX-A

COX is a small, cytolytic virus belonging to the Enterovirus group of Picornaviridae family. It possesses a positive single-stranded RNA genome, lacks an envelope, and features an icosahedral capsid with surface viral proteins (79). COX is classified into two groups: 1) coxsackievirus A (COX-A), with 23 serotypes commonly linked with hand, foot, and mouth disease and 2) coxsackievirus B (COX-B), with six serotypes often associated with myocarditis, among other conditions (80).

COX viruses, including COX-A21 and COX-B3, have been involved in OVT (81). COX-A21 stands out as a great candidate for several reasons. First, it boasts a highly specific and efficient ligand-receptor system for cellular entry. It binds decay-accelerating factor on the cell surface and requires the concurrent presence of intercellular adhesion molecule-1 for viral infection, facilitating the entry of the OVs into tumor cells (82). The replication of COX-A depends on nuclear factor κB (83). Subsequently, infected host cells undergo apoptosis induced by COX-A or a T-cell immune response. Clinical data show the safety of COX-A21, with no reported grade 3 or 4 AEs (84).

A bioselected COX-A21 strain named V937, without any modification, was used (85). V937 infects and leads to direct lysis of tumor cells that overexpresses intercellular adhesion molecule-1 (ICAM01). In the latest phase II open-label clinical trial in 2023, injection of V937 showed antitumor activity with a decrease in the size of injected and non-injected liver tumor cells metastases from melanoma. the clinical efficiency and safety of V937 were tested, with no patients reaching complete response or partial response. PFS was observed in all patients, with a median PFS of 3.7 months and a PFS rate of 9% at week 26 (86). Although V937 demonstrated relative safety in human participants, its efficacy in OVT warrants further investigation.

Later in 2024, a preclinical study further investigated the role of V937 alone versus V937 combined with pembrolizumab therapy in the treatment of HCC (87). When V937 was injected into non-contact tumor cell lines, a significant increase in IFN-α, IL-12, IFN-γ, IP-10, macrophage inflammatory protein (MIP)-1α, and IL-6 was observed. Pembrolizumab induces the expression of ICAM-1 on the surface of tumor cells, leading to increased infection and attack of V937 on HCCs, thereby establishing an antitumoral effect.

In addition to its use in liver tumors, COX-A has been mainly used for melanoma (88), and has shown some effectiveness in colorectal cancer (89), small cell lung cancer (90), etc.

### 3.5 IV

IV, a negative-sense single-stranded RNA virus from the Orthomyxoviridae family, exhibits a pleomorphic virion measuring 100–120 nm in diameter, encapsulated within a spherical bilayer envelope (91). IV comprises 7 serotypes, with IV A and IV B being the most commonly spread. The envelope surface of IV bears >500 spike-like projections, comprised predominantly of the glycoproteins hemagglutinin and neuraminidase in a 10 to 1 ratio (92). Upon entering the host cell, hemagglutinin undergoes activation by serine proteases. IV then integrates into the host genome, regulated by NS1, which acts as an interferon antagonist during virus replication (93).

IV can elicit a robust cytokine response, activating the adaptive immune system, and further promoting cytokine secretion. This potent ability to induce host cell death positions IV as one of the most commonly used OVs in cancer therapy (94). However, all studies remain in the realm of basic science research, with no recent clinical studies conducted.

Similar to oHSV, PD-L1 antibodies were incorporated into IV to target HCC cells (95). This oncolytic IV was identified through screening in pathogen-free chicken embryos, with all eight plasmids containing IV A/Puerto Rico/8/34 (PR8) and wild-type PR8 viral genetic materials. These plasmids were then recombined with the heavy and light chains of the PD-L1 antibody gene, named rgFlu/PD-L1. In cell culture experiments infecting normal MIHA liver cells and HCC cells, rgFlu/PD-L1 significantly reduced the viability of all tested HCC cells, with host cell survival rates decreasing as the duration and dose of infection increased. Importantly, normal MIHA cells remained unaffected, demonstrating the specificity of IV in targeting HCC cells exclusively. During infection, PD-L1 expression levels were suppressed, and apoptosis increased in rgFlu/PD-L1-treated HCC cells. In the mouse model, tumor size and weight significantly decreased compared with the control group 32-d post-injection, indicating the potential of rgFlu/PD-L1 for improving long-term survival rates (95). Safety assessments revealed negligible impact on organs, other than induced necrosis in HCC cells in the liver. The mechanism of HCC cell elimination by rgFlu/PD-L1 involved enhancing the activity and infiltration of CD8+ T cells and dendritic cells via the cyclic GMP-AMP synthase stimulator of interferon genes pathway, evidenced by the elevated levels of STING, phosphorylated STING, IRF3, phosphorylated IRF3, and TANK-binding kinase 1.

Cytotoxic T lymphocyte-associated antigen 4 (CTLA4) is an inhibitory regulator of T cells that tumors often employ to evade the immune system (96). An anti-CTLA-4 antibody was integrated into IV to evaluate its efficacy in HCC cells (93). The heavy and light chains encoding the CTLA4 antibody with PR8 IV yielded the recombinant OV named rFlu-huCTLA4 through reverse transcription. The TCID_50_ was 8-9 LogTCID_50_/ml. Cell viability assessments conducted 48, 72, and 96 h after rFlu-huCTLA4 injection into MIHA and HCC cell lines revealed unaffected, whereas HCC cell death increased proportionally with dose and duration of exposure. Moreover, the apoptosis rate was significantly higher in HCC cells (26.76%) than MIHA cells (3.45%) (93). In a mouse model, infection with rFlu-huCTLA4 increased the number of CD8+ T cells by 23.9%, targeting and eliminating HCC cells and CD4+ T cells by 38.7%. Liver tumor size and weight were significantly smaller compared with those in the MIHA-treated group. rFlu-huCTLA4. No virus was detected in other organs 40 d post-treatment.

Despite promising preclinical findings, no clinical studies utilizing IV as a potential OVT for liver tumor have been publicly available in the past five years. Limited studies have shown the implication of IV in pancreatic ductal adenocarcinoma (97), lung tumors (98), etc.

## 4 Discussion and future directions

Researchers have used various kinds of OVs with different modifications to understand their mechanisms and test their efficacy on liver cancer. However, there is still significant room for optimizing the treatment outcomes of OVs in the future.

The route of administration for OVs should be optimized based on the stage of liver cancer and the type of OV used. For example, patients with liver metastases may not respond well if OVs are injected intratumorally due to the difficulty of injecting multiple tumors and the risk of injections near important anatomical structures such as biliary structures (99, 100). Systemic delivery, such as IV injection and hepatic arterial injection (HAI), would be more effective options as they can distribute OVs throughout the entire body, not just within the liver tumor (100). The increased survival rates had been shown in patients with liver metastases via OV treatment with HAI, compared with OV treatment intratumorally (101). However, the quantity of virus delivered over a long pathway may be compromised by neutralizing antibodies (99). Local liver tumors are more responsive to IT and intralesional injection, which helps avoid the barrier of the extracellular matrix (102). Bacterial collagenase could be used to increase OV infiltration for local tumors (103).

Despite the effectiveness and benefits of OVs in liver tumor treatment, several barriers need to be solved. First, patients with HCC often present with underlying liver cirrhosis and dysfunction, making them more susceptible to adverse effects from OVs, which can lead to liver toxicity (99). Second, the number of studies (including preclinical and clinical) specifically focused on liver OVT is limited and has not demonstrated significant clinical effectiveness of OVs (104). Third, the evaluation of antitumor activity could be improved. For instance, many studies rely solely on changes in tumor size to assess OVT effectiveness, overlooking changes in tumor density and molecular markers of tumor necrosis, such as immune cell infiltration (99).

While conventional approaches or OVT alone may not achieve superior efficacy in liver tumor treatment due to tumor heterogeneity, combining these two approaches has proven to be effective in liver cancer treatment (105). Pathways targeted by small molecular-based drugs for liver cancer treatment target sometimes overlap with those targeted by OVT, such as the EGF pathway (101). Transarterial chemoembolization (TACE) can increase tumor response during the treatment, but the antitumor effect often diminishes shortly after the treatment. However, when combined with OVs, TACE can directly deliver OVs through the blood vessels, avoiding attacks on OVs by the host immune response, which prevents a decrease in OV concentration and avoids AE on other parts of the body (105). Chemotherapy usually has limited effect on liver tumors due to the presence of resistant disease and liver toxicity. Conversely, liver cancer cells are less resistant to OVs, and OVs cause lower toxicity to the liver. Clinical research has shown increased treatment outcomes using this combined approach (106). For example, when oHSV is used with cisplatin in HCC, cytotoxicity increased in all cell lines tested (107).

## 5 Conclusion

The landscape of OVs in cancer treatment shows promising strides, but their application in liver cancer treatment faces a significant gap between preclinical promise and clinical validation. Although basic science studies offer encouraging insights, the lack of robust clinical evidence leaves a critical void in understanding their effectiveness in treating liver cancer. Although these viruses often demonstrate a favorable safety profile, it is crucial to recognize that this observation might be skewed by small sample size and the selective withdrawal of patients with severe illness.

To truly harness the potential of OVs in liver cancer treatment, extensive clinical investigation is imperative. Larger-scale clinical trials are necessary to provide concrete evidence of efficacy and safety in real-world patient populations. Bridging this gap between basic science research and clinical application is essential for validating OVs as an effective therapeutic option for patients with liver cancer. This journey toward clinical validation not only enhances our understanding of innovative treatments, but also holds the promise of improving outcomes for patients with liver cancer.
